# The Impairment of MAGMAS Function in Human Is Responsible for a Severe Skeletal Dysplasia

**DOI:** 10.1371/journal.pgen.1004311

**Published:** 2014-05-01

**Authors:** Cybel Mehawej, Agnès Delahodde, Laurence Legeai-Mallet, Valérie Delague, Nabil Kaci, Jean-Pierre Desvignes, Zoha Kibar, José-Mario Capo-Chichi, Eliane Chouery, Arnold Munnich, Valérie Cormier-Daire, André Mégarbané

**Affiliations:** 1 Unité de Génétique Médicale et Laboratoire International associé INSERM à l'Unité UMR_S 910, Faculté de Médecine, Université Saint-Joseph, Beirut, Lebanon; 2 Département de Génétique, Unité INSERM U781, Université Paris Descartes-Sorbonne Paris Cité, Fondation Imagine, Hôpital Necker Enfants Malades, Paris, France; 3 University of Paris-Sud, CNRS, UMR 8621, Institute of Genetics and Microbiology, Orsay, France; 4 Inserm, UMR_S 910, Marseille, France; 5 Aix Marseille Université, GMGF, Marseille, France; 6 Center of Excellence in Neuroscience of Université de Montréal, Centre de Recherche du CHU Sainte-Justine, Montréal, Canada; 7 Department of Obstetrics and Gynecology, Université de Montréal, Montréal, Canada; Max Planck Institute for Molecular Genetics, Germany

## Abstract

Impairment of the tightly regulated ossification process leads to a wide range of skeletal dysplasias and deciphering their molecular bases has contributed to the understanding of this complex process. Here, we report a homozygous mutation in the mitochondria-associated granulocyte macrophage colony stimulating factor-signaling gene (*MAGMAS*) in a novel and severe spondylodysplastic dysplasia. MAGMAS, also referred to as PAM16 (presequence translocase-associated motor 16), is a mitochondria-associated protein involved in preprotein translocation into the matrix. We show that *MAGMAS* is specifically expressed in trabecular bone and cartilage at early developmental stages and that the mutation leads to an instability of the protein. We further demonstrate that the mutation described here confers to yeast strains a temperature-sensitive phenotype, impairs the import of mitochondrial matrix pre-proteins and induces cell death. The finding of deleterious *MAGMAS* mutations in an early lethal skeletal dysplasia supports a key role for this mitochondrial protein in the ossification process.

## Author Summary

Skeletal dysplasias (SD) refer to a complex group of rare genetic disorders affecting the growth and development of the skeleton. The identification of the molecular basis of a considerable number of SD has greatly expanded our knowledge of the ossification process. Among SD, spondylodysplastic dysplasia is a generic term describing different conditions characterized by severe vertebral abnormalities and distinct by additional specific features. Several entities within this group are well defined. However, a few cases remain unclassified, of which a novel autosomal recessive spondylometaphyseal dysplasia recently reported by Mégarbané et al. in two Lebanese families. Here, we identified *MAGMAS* as a candidate gene responsible for this severe SD. MAGMAS, also referred to as PAM16, is a mitochondria-associated protein, involved in pre-proteins import into mitochondria and essential for cell growth and development. We demonstrated that *MAGMAS* is expressed in bone and cartilage in early developmental stages underlining its specific role in skeletogenesis. We also give strong evidence of the deleterious effect of the identified mutation on the *in-vivo* activity of Magmas and the viability of yeast strains. Reporting deleterious *MAGMAS* mutation in a SD supports a key and specific role for this mitochondrial protein in ossification.

## Introduction

During embryonic development and postnatal growth period, most longitudinal bone growth occurs via endochondral ossification, a process in which chondrocytes produce a cartilage template that will ultimately be replaced by bone [1]. Disturbance of this tightly regulated process results in skeletal dysplasias (SD), which are an extremely diverse and complex group of rare genetic diseases [2]. Causative mutations have now been identified in over 230 different genes in more than 400 unique skeletal phenotypes [3], [4]. However, the genetic basis of over 100 different entities remains to be determined. Among SD, spondylodysplastic dysplasia is a generic descriptive term for a heterogeneous group of SD characterized by severe vertebral abnormalities and distinctive skeletal or extra-skeletal features [3], [4]. The outcome is variable from early lethality to long-term survival. The last international classification recognizes at least five distinct entities within this group of rare disorders, i) achondrogenesis type 1A [MIM 200600] due to mutations in *TRIP11* [MIM 604505], which encodes the thyroid hormone receptor interactor 11, ii) Schneckenbecken dysplasia [MIM 296250] due to mutations in *SLC35D1* [MIM 610804], encoding the solute carrier family 35 member D1, also known as UDP-Galactose transporter-related 7 iii) spondylometaphyseal dysplasia (SMD) Sedaghatian type [MIM 250220], a heterogeneous condition that is due in some cases, to mutations in *SBDS* [MIM 607444] involved in the processing of ribosomal RNA, iv) fibrochondrogenesis [MIM 228520], due in some cases to *COL11A1* mutations and v) opsismodysplasia [MIM 258480], due to mutations in *INPPL1* [MIM 600829] that encodes the inositol polyphosphate phosphatase like-1 [5]. The wide range of proteins involved in this group of SD points out the complexity of the ossification process.

Although different entities within the group of spondylodysplastic dysplasia are well defined, a few cases remain unclassified. Recently, Mégarbané et al. have reported on two unrelated consanguineous Lebanese families with four affected cases presenting a novel type of early lethal spondylometaphyseal dysplasia [6], [7]. To elucidate the molecular basis of this entity, we performed exome sequencing in these two distinct families and identified a homozygous mutation in *MAGMAS* (Mitochondria-associated granulocyte macrophage colony stimulating factor-signaling molecule) in patients from both families.

## Results

### Patients

**Figure 1:**
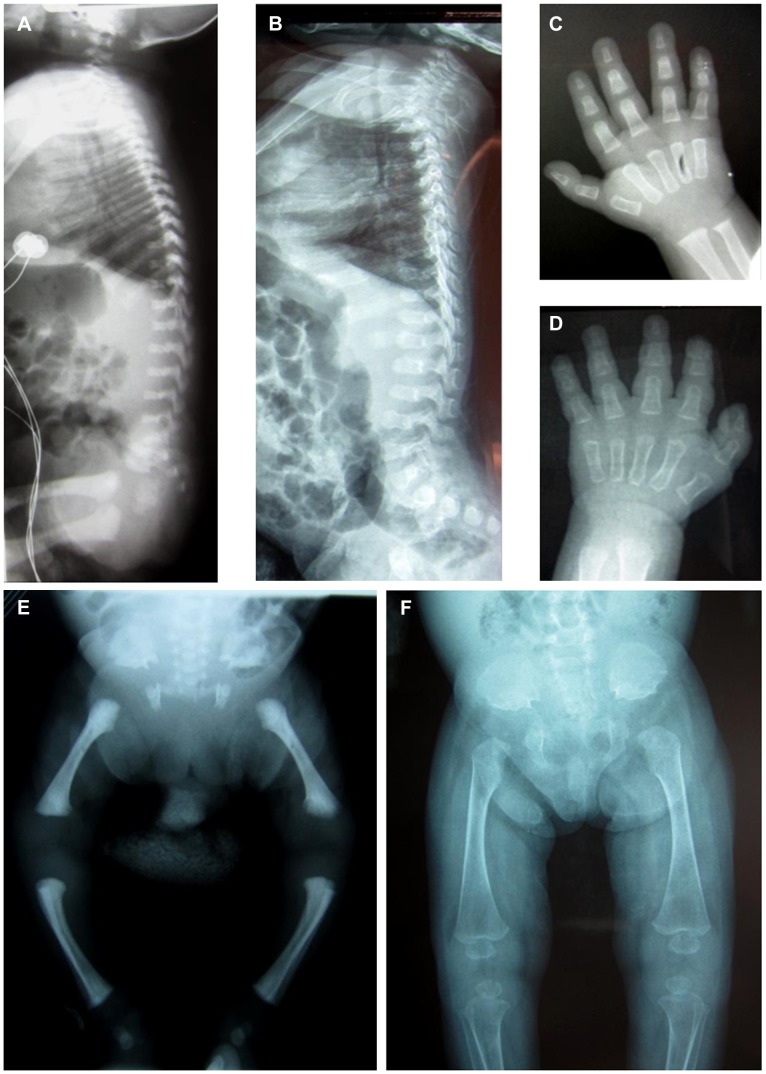
Radiological features of the patients F2-IV.3 and F1-IV.3. Radiographs of patient **F2-IV.3** at 9 months (**A, C, E**) and **F1-IV.3** at birth (**B, F**) and at 3 months (**D**) show platyspondyly, square iliac bones, and delayed epiphyseal ossification.

**Figure 2:**
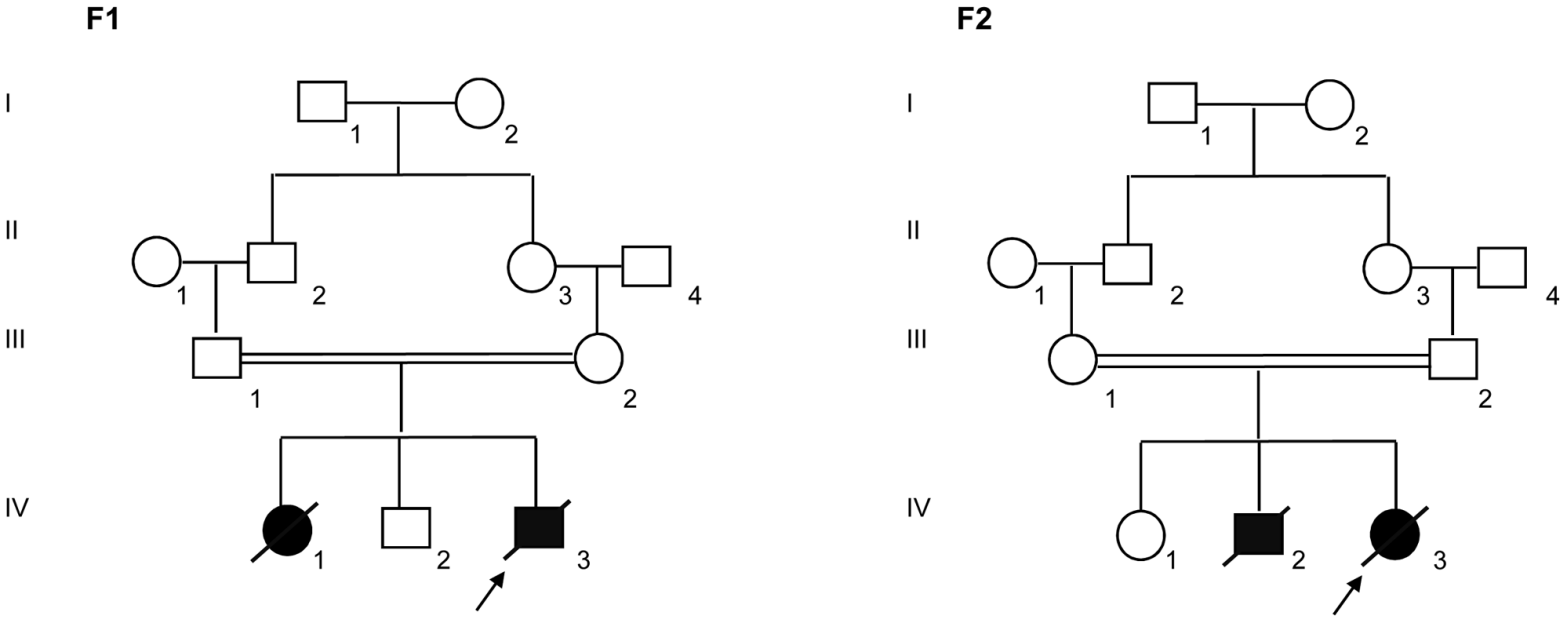
Pedigrees of the families F1 and F2 included in this study. Blackened symbols indicate affected individuals.

Two unrelated consanguineous Lebanese families with four affected cases presenting a novel type of early lethal spondylodysplastic dysplasia, recently reported by Mégarbané et al. [6], [7], were included in this study. The main clinical features were pre- and postnatal growth retardation, developmental delay, dysmorphic features, prominent abdomen, narrow thorax with short ribs and short limbs. Skeletal features included severe platyspondyly at birth that improved somewhat over time, square iliac bones, horizontal acetabulae, short long bones with abnormal modeling, widening of the distal femoral metaphyses and delayed epiphyseal ossification (Figure 1). In the first reported family (Family F1, Figure 2) [6], both affected sibs died at an early age from respiratory insufficiency (F1-IV.1 at age 9 months and F1-IV.3 at age 2 years) while heart failure was the cause of the early death, at age 2 years, in patients (F2-IV.2 and F2-IV.3) of the second family (Family F2: individuals IV.2 and IV.3) [7] (Figure 2).

### Molecular findings

Exome sequencing has been performed in Family 2, in three individuals: the proband (F2-IV.3) and her two unaffected parents (F2-III.1 and F2-III.2) (Figure 2). Considering the recessive inheritance of the disease in the pedigree and consanguinity (F = 1/16), we assumed identity by Descent [8]. Using an in-house bioinformatics tool, we realized identity by descent filtering of exome data and identified 1037 homozygous variants in the proband, which were also heterozygous in both parents. Of these 1037 variants, 916 were exonic and/or splice site mutations, of which 434 were either non-synonymous and nonsense single nucleotide variants, or small insertions/deletions leading to a shift in the reading frame.

In order to further refine the list of candidate nucleotide variations, additional filtering was performed on what we called the “dbSNP13700” database (i.e. dbSNP137, from which we have removed mutations that are reported as pathogenic alleles, as well as all referenced variants with frequencies lower than 1%, or without any frequency information). Additional filtering was also performed using our in-house exome database that includes all homozygous variants detected by exome sequencing of 14 Lebanese unaffected individuals. We ended up with a list of 16 candidate homozygous by descent variants in the proband F2-IV.3.

In family 1, exome sequencing has been performed only in the proband (F1-IV.3, Figure 2). Using the same analysis pipeline and after all filtering steps, we were able to identify 59 homozygous variants in the genes coding regions of this patient.

**Figure 3:**
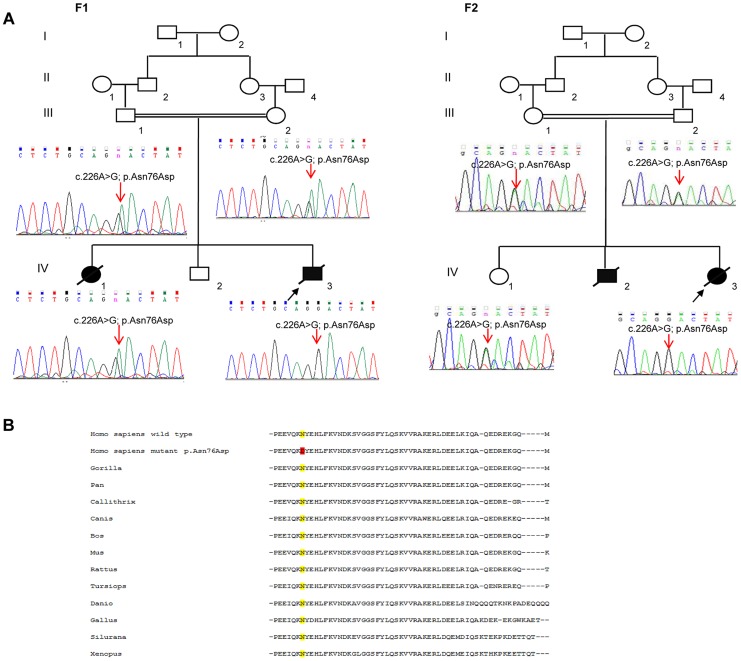
The missense *MAGMAS* mutation c.226A>G (p.Asn76Asp). (**A**) Sequencing chromatograms showing the segregation of the c.226A>G transition in *MAGMAS* with the disease in both families F1 and F2. n: heterozygous peak. (**B**) Multiple alignments between human MAGMAS protein and several orthologs showing that the Asparagine at codon 76 in MAGMAS is well conserved among mammals and vertebrates.

In total, patients F1-IV.3 and F2-IV.3 shared 13872 homozygous variants, of which only 3 exonic variations were both homozygous by descent in the proband from family 2 (F2-IV.3), and homozygous in the proband from family 1 (F1-IV.3), after applying the different filters (Table S1). Of those three variants, only one missense mutation (NM_016069: c.226A>G; p.Asn76Asp) in the *PAM16* gene (presequence translocase-associated motor 16), that is more commonly referred to as *MAGMAS* (Mitochondria-associated granulocyte macrophage CSF-signaling molecule), was predicted to have pathogenic effects using different bioinformatics prediction softwares, such as: Mutation Taster (disease causing variation with a probability value of 0.99999957), SIFT (damaging effect, score 0.006) [9], PROVEAN (deleterious effect, score −4.021) [10] and PolyPhen-2 (possibly damaging effect, score 0.728 HumDiv). By Sanger sequencing, we confirmed the segregation of the c.226A>G transition in *MAGMAS* with the disease in both families (Figure 3A): the mutation was homozygous in the patients, heterozygous in the parents and in the unaffected sibs in both families. We also demonstrated that the variation was absent in a set of 550 Lebanese control chromosomes. Protein sequence alignments showed that the Asparagine at codon 76 in MAGMAS is well conserved among mammals and vertebrates (Figure 3B).

The two patients described here are not related. However, they display the same homozygous missense mutation, thus suggesting the existence of a founder mutation. In order to verify this hypothesis, we used both exome data and genotyping of STR markers. By analyzing exome data, we were able to identify 11 regions of shared homozygosity on chromosome 16. However, only three regions displayed a size over 100 kb, of whom two were containing or contiguous to the *MAGMAS* locus (Chr16: 4015729–7102036 (∼3 Mb) and Chr16: 8728764–8840025 (∼1.1 Mb)). Also, by using PLINK [11], we were able to reconstruct haplotypes from exome data of both patients, and here again, we identified a 3.6 Mb shared homozygous region located between SNP markers rs3730119 and rs4616299 on chromosome 16p13.3 (Chr16:4,057,603–7,657,432). Genotyping of microsatellite markers allowed to refine this region and define a minimal ancestral common homozygous haplotype spanning 1.9 Mb between markers D16S758 and D16S243 containing *MAGMAS* (Figure S1). Altogether, our results demonstrate that the c.226A>G transition in *MAGMAS* (p.Asn76Asp) is likely to be pathogenic in both families. *MAGMAS* is located on chromosome 16p13.3 and is composed of five exons. It encodes MAGMAS, an essential component of the pre-sequence translocase-associated protein import motor (PAM) that regulates pre-protein translocation into the mitochondrial matrix [12].

### Gene expression analysis

**Figure 4:**
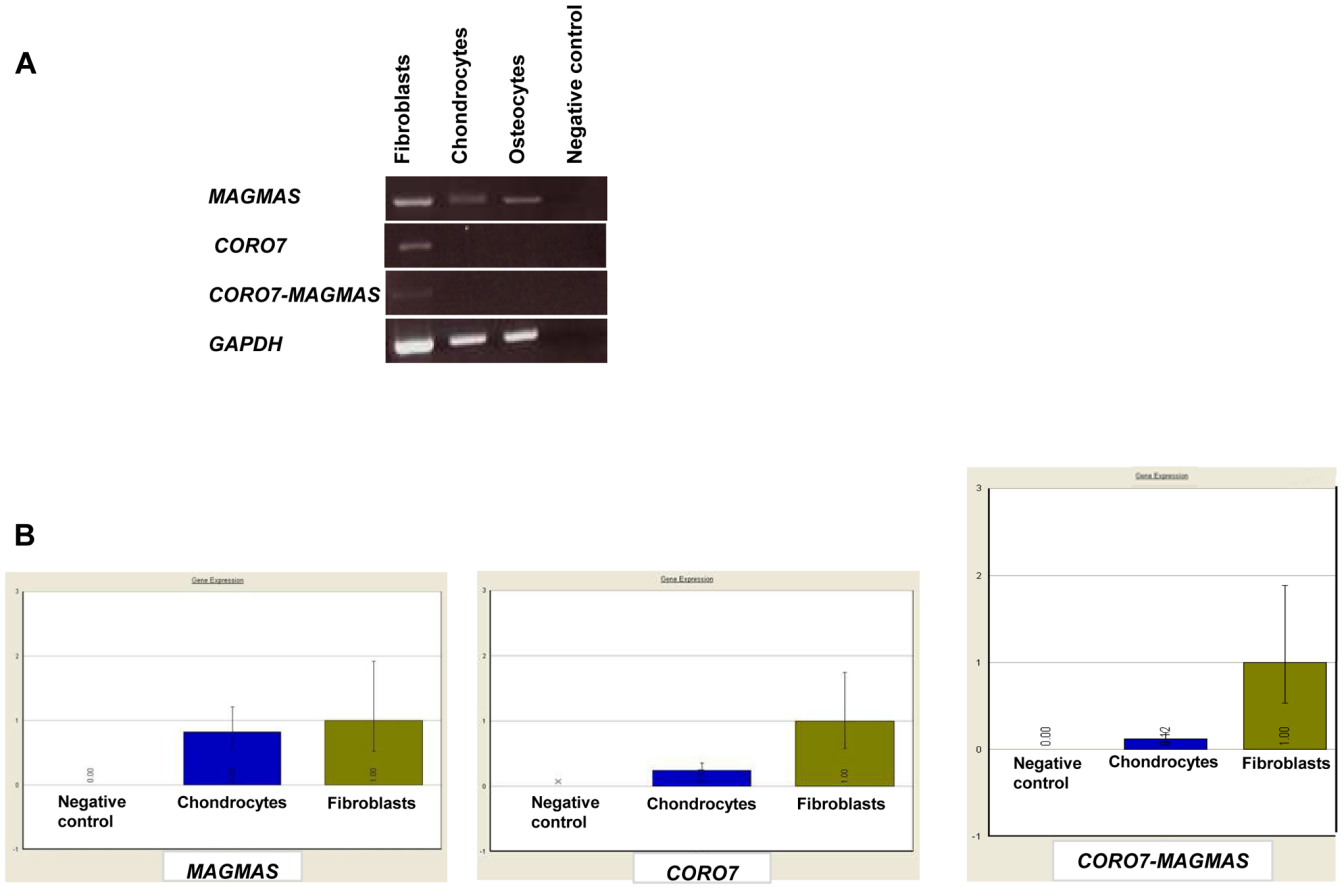
Transcript expression analysis of *MAGMAS*, *CORO7* and *CORO7-MAGMAS*. (A) Expression analysis of *MAGMAS*, *CORO7* and *CORO7-MAGMAS* by RT-PCR in control fibroblasts, chondrocytes and osteoblasts, showing that, contrary to *MAGMAS*, neither *CORO7* nor *CORO7-MAGMAS* was significantly expressed in chondrocytes and osteoblasts. (**B**) Quantitative expression analysis of *MAGMAS*, *CORO7* and *CORO7-MAGMAS* showing a very low level of expression of *CORO7* and *CORO7-MAGMAS* in chondrocytes compared to that of *MAGMAS* in the same sample.

A naturally occurring read-through transcription between the neighboring *CORO7* (coronin 7) and *MAGMAS* genes on chromosome 16 is stated in the Genome UCSC browser (NM_001201479). To determine if the fusion protein is involved in the pathogenesis of the severe skeletal manifestations observed in the affected cases, we checked the expression of *CORO7*, *MAGMAS* and *CORO7-MAGMAS* fusion transcript in human cultured control cells (fetal chondrocytes, osteoblasts and skin fibroblasts) by Reverse Transcriptase PCR. *CORO7*, *MAGMAS* and *CORO7-MAGMAS* were all expressed in skin fibroblasts. *MAGMAS* expression in bone and cartilage was confirmed. On the other hand, neither *CORO7* nor *CORO7-MAGMAS* was expressed in significant amounts in chondrocytes and osteoblasts (Figure 4A). This result was confirmed by quantitative real-time RT-PCR (Figure 4B): The ratio of *MAGMAS* in chondrocytes versus in fibroblasts is 0.82 whereas the ratios of *CORO7* and *CORO7-MAGMAS* in chondrocytes versus in fibroblasts are 0.24 and 0.12, respectively.

### Immunolocalization of MAGMAS protein in wild type growth plates

**Figure 5:**
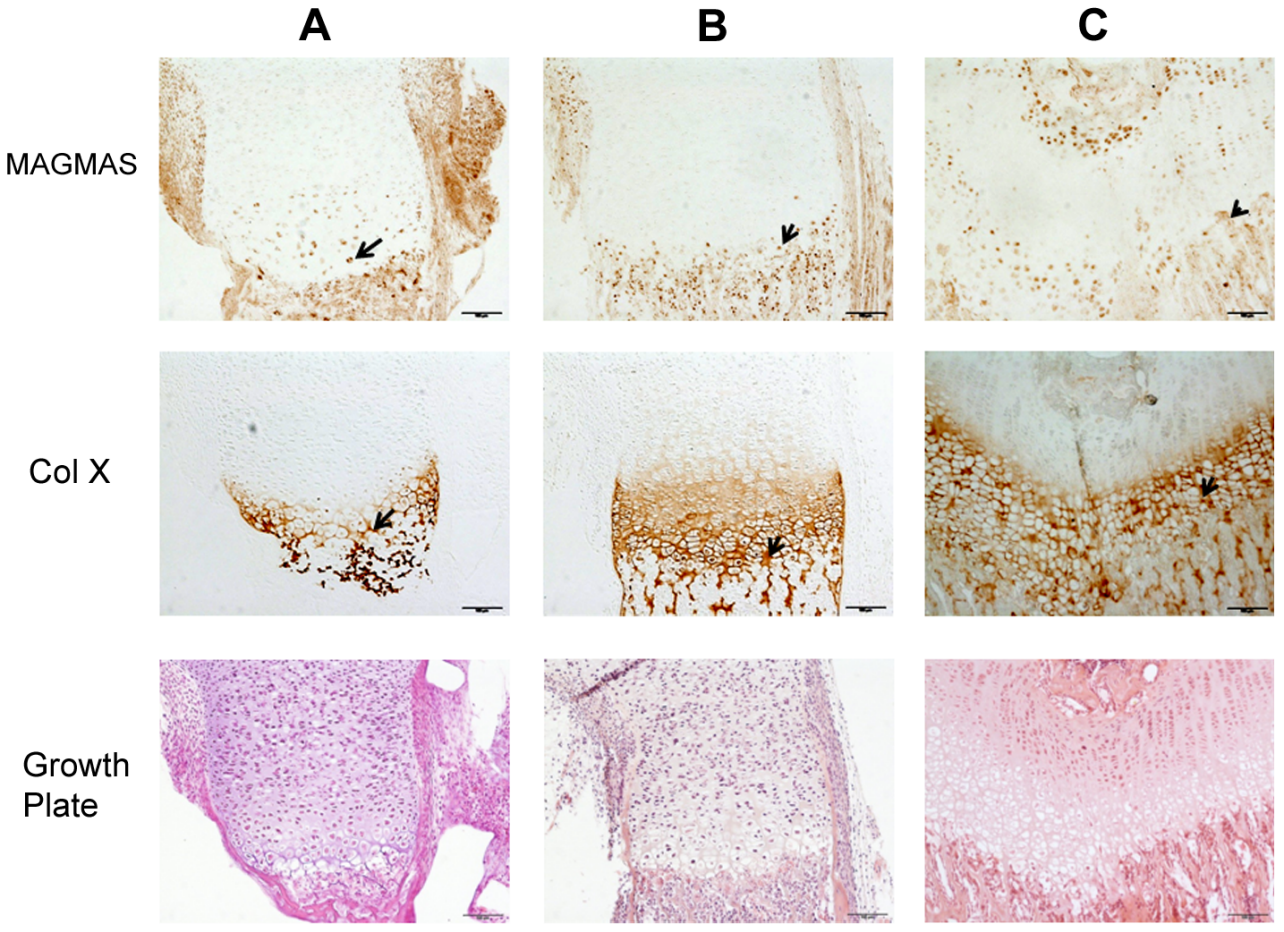
MAGMAS localization in control growth plates of mice. Distal femurs of wild-type mice at different developmental stages; (**A**) 16.5-day embryo; (**B**) Newborn and (**C**) 2 week-old; were fixed and stained with anti-MAGMAS antibodies. Sections were also stained with anti-CoX (marker of differentiated hypertrophic chondrocytes) and with HES. MAGMAS expression is reflected by the brown precipitate resulting from the peroxidase reaction. MAGMAS was detected mainly in hypertrophic chondrocytes (black arrows) and osteocytes of control growth plates at the different developmental stages. Its expression was also detected in some type X collagen –negative chondrocytes that are initiating the process of differentiation.

To further confirm the specific involvement of MAGMAS in skeletogenesis, we performed immunohistochemical staining of fixed femurs isolated from wild type (WT) mice at different developmental stages from E16.5 to 2 weeks of age. Type X collagen staining was also performed in order to mark the terminally differentiated hypertrophic chondrocytes. MAGMAS protein was expressed in bone and cartilage. Expression of the protein was mostly observed in the hypertrophic zone of the cartilage mainly at postnatal ages and in the primary spongiosa of trabecular bone. Its expression in proliferative chondrocytes and in the resting zone of the cartilage was not detected (Figure 5).

### Functional complementation

Human and murine forms of *MAGMAS* present a very high degree of homology. Of the 125 amino acids comprising the mature protein, 120 are identical [13]. The evolutionary conservation of *MAGMAS* from yeast to mammals [14] and the lethal phenotype of its deletion (*pam16Δ*) in *Saccharomyces cerevisae* [15] and *Drosophila* [16] imply a fundamental role of this protein in cell growth and development. Previous studies have shown that human *MAGMAS* exhibits a complete growth complementation of deleted *pam16* yeast cells at all temperatures [17].

**Figure 6:**
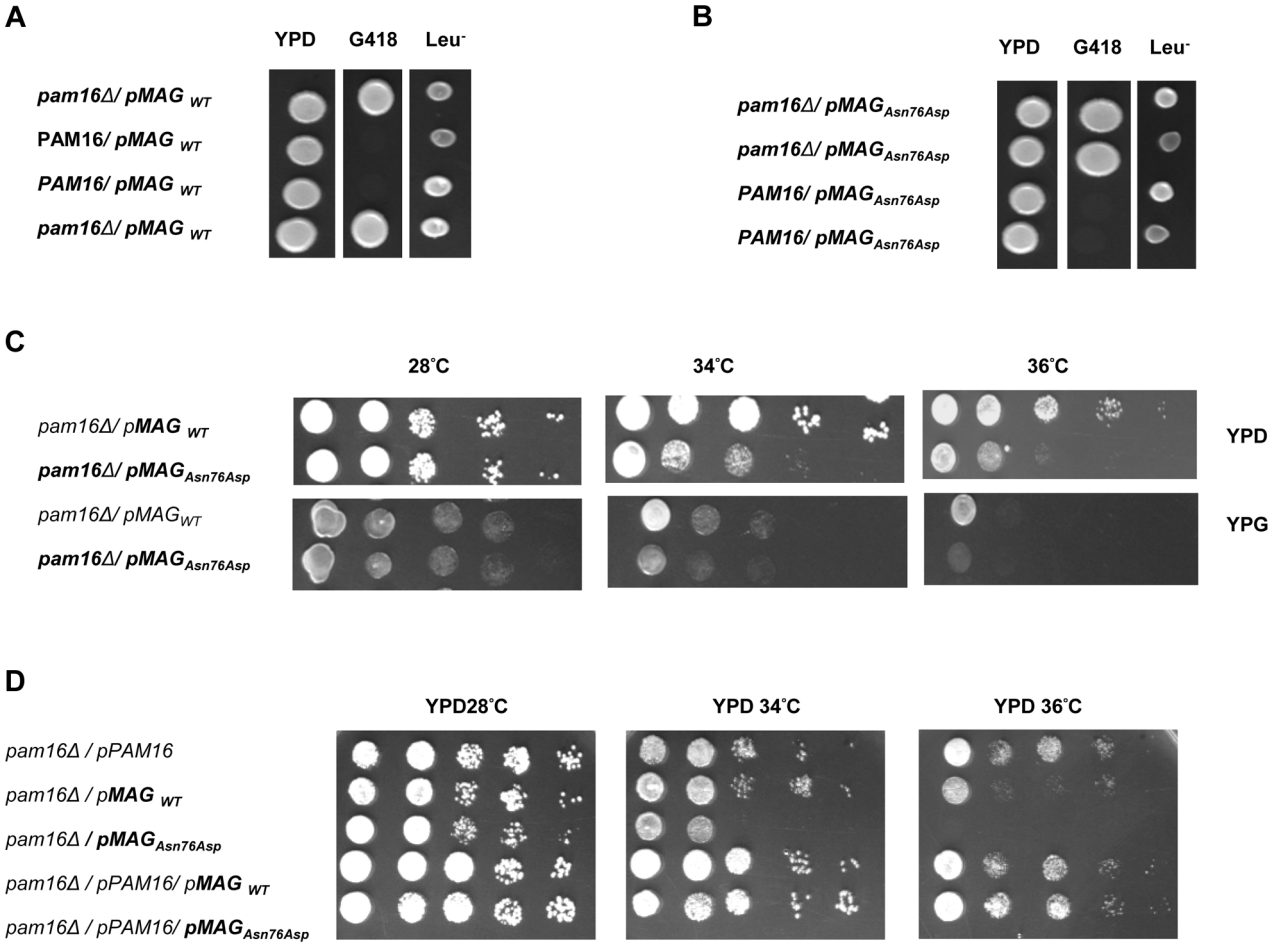
*In vivo* growth analysis. (**A–C**) Diploid BY4743*Δlip3*/+ cells were transformed with (**A**) *pRS415-WT-MAGMAS* and (**B**) *pRS415-Asn76Asp-MAGMAS*, and then subjected to sporulation followed by tetrad dissection analysis. Spores from a single ascus grown on YPD were spotted onto: G418 for the selection of spores carrying the deleted *pam16* gene (*pam16Δ::kanMX6*) and onto Leu drop-out media for the selection of *MAGMAS* positive spores. (**C**) Growth complementation analyses showing the temperature-sensitive growth phenotype of the mutant pam16Δ-MAG***_Asn76Asp_*** in the BY4743 genetic background at 34°C (307.15°K) and 36°C (309.15°K) on both fermentative (YPD) and non-fermentative (YPG) media. (**D**) Haploid YPH499 *pam16Δ/pPAM16* cells transformed with *pRS415-WT-MAGMAS* and *pRS415-Asn76Asp-MAGMAS* were tested for their ability to rescue the inviability of the *pam16Δ* strain by plasmid shuffling at different temperatures.

In order to obtain *in vivo* evidence of the deleterious nature of the identified mutation, we performed a yeast complementation assay using genetic approaches as described previously by Sinha et al. [17]. We first found that at 28°C (301.15°K), both WT and mutant *MAGMAS* genes were able to complement the growth defect of the *pam16Δ* cells since spores expressing *MAGMAS_WT_* and *MAGMAS_Asn76Asp_* were viable when spotted onto G418 selective medium (Figure 6A, 6B). To figure out if the Asn76Asp mutant confers a deleterious growth phenotype at non-permissive temperatures, tenfold serial dilutions of *pam16Δ* spores expressing WT or mutant *MAGMAS* were subsequently spotted onto fermentable (YPD) and non-fermentable (YPG) growth media and incubated at different temperatures for 3 days. As shown in Figure 6C, the mutant strain *pam16Δ*-MAG_Asn76Asp_ that presented a normal growth phenotype on both YPD and YPG media at 28°C (301.15°K), grew more slowly than *pam16Δ-*MAG_WT_ on YPD at 34°C (307.15°K) and 36°C (309.15°K). A more severe growth defect of *pam16Δ-*MAG_Asn76Asp_ on YPG was evidenced by a very slow-growth at 34°C (307.15°K), and inviability at 36°C (309.15°K). To confirm this temperature-sensitive growth phenotype, we secondly tested the ability of *MAGMAS_Asn76Asp_* to rescue the inviability of the *pam16Δ* strain by plasmid shuffling using the haploid YPH499*pam16Δ* strain transformed with a plasmid encoding the yeast WT *PAM16* (y*PAM16*). Unlike WT *MAGMAS* expressing strains, the mutant *pam16Δ*-MAG_Asn76Asp_ exhibited an obvious slow-growth at 34°C and was unable to grow at 36°C (309.15°K) on YPD (Figure 6D). On the other hand, *pam16Δ*-*PAM16*-MAG_Asn76Asp_ cells carrying a WT copy of y*PAM16* in addition to the mutant *MAGMAS* expressing plasmid, showed a normal growth phenotype at all temperatures.

### Protein import and MAGMAS expression in yeast extracts

**Figure 7:**
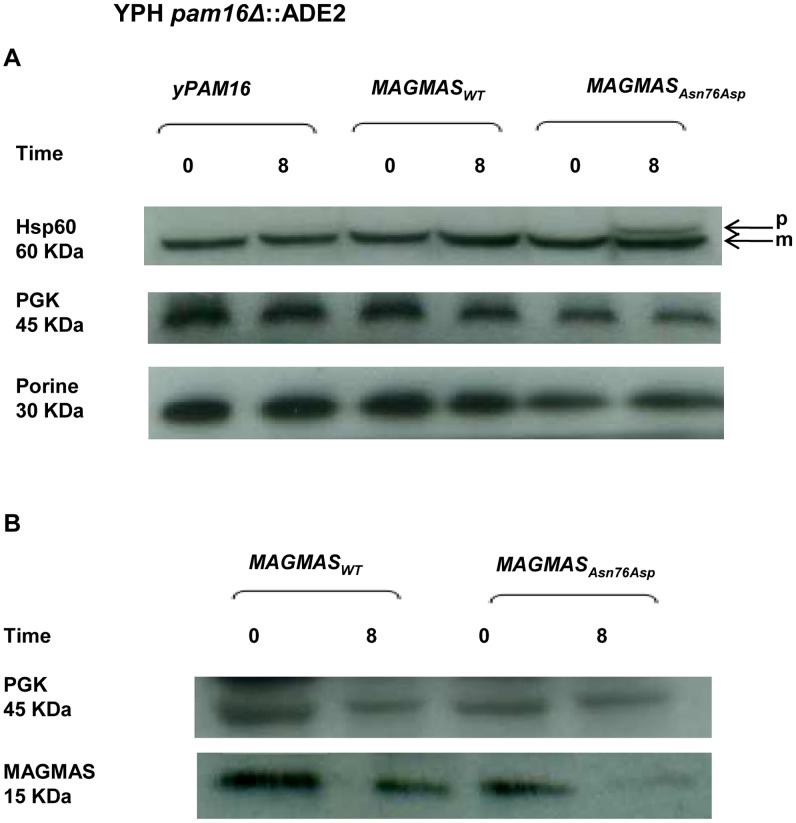
Preprotein translocation and MAGMAS expression analyses in yeast cells. *pam16Δ*-MAG_WT_ and *pam16Δ*-MAG***_Asn76Asp_*** strains in the YPH499 genetic context, expressing wild-type and mutant *MAGMAS*, accordingly were constructed by plasmid shuffling (see Materials and Methods). Strains were grown at 28°C (301.15°K) then shifted for 8 h (28800 s) at 37°C (310.15°K) to induce the mutant phenotype. (**A**) Analysis of protein translocation in yeast cells: Non-processed precursor form and mature Hsp60 were detected by Western blotting using anti-Hsp60 antibodies. The non-processed Hsp60 precursor accumulated in the mutant strain after induction of the phenotype. Only the mature form of Hsp60 was detected in wild-type *yPAM16* and *MAGMAS* expressing yeast cells. PGK was used as a loading control and Porin as a mitochondrial loading control. p: precursor form of Hsp60, m: mature form of Hsp60. (**B**) Immunoblotting using Human anti-MAGMAS antibodies detected a lower level of MAGMAS protein in the mutant strain when shifted at non-permissive temperature, compared to the amount in *MAGMAS_WT_* expressing strains.

MAGMAS is an essential component of PAM, a peripheral mitochondrial inner membrane complex, that is crucial for preprotein translocation into the mitochondrial matrix [12], [18]. Preproteins targeted to the matrix cross the outer mitochondrial membrane through the TOM complex then pass through the inner membrane via the TIM23^MOTOR^ complex which is composed of the TIM23 translocase associated with the PAM complex [19] (Figure S2). To assess the transport function of the mutant MAGMAS_Asn76Asp_, we evaluated the protein translocation activity by following the mitochondrial import of the Hsp60 protein. Non-processed mitochondrial Hsp60 was accumulated in significant amounts in the *pam16Δ*-MAG_Asn76Asp_ strain when the mutant phenotype was induced (37°C, 310.15°K), but was undetectable in WT *pam16Δ-PAM16* and *pam16Δ-MAGMAS* expressing cells (Figure 7A). We then evaluated the expression levels of MAGMAS protein in the different strains, by performing a Western blot using Human anti-MAGMAS antibodies. Immunoblotting detected a lower level of mutant MAGMAS when the strain was shifted at the non permissive temperature, compared to the amount of MAGMAS_WT_ in the same condition (Figure 7B).

### Mitochondrial morphology and peroxisomal biogenesis defects in the MAGMAS_Asn76Asp_ expressing strain

**Figure 8:**
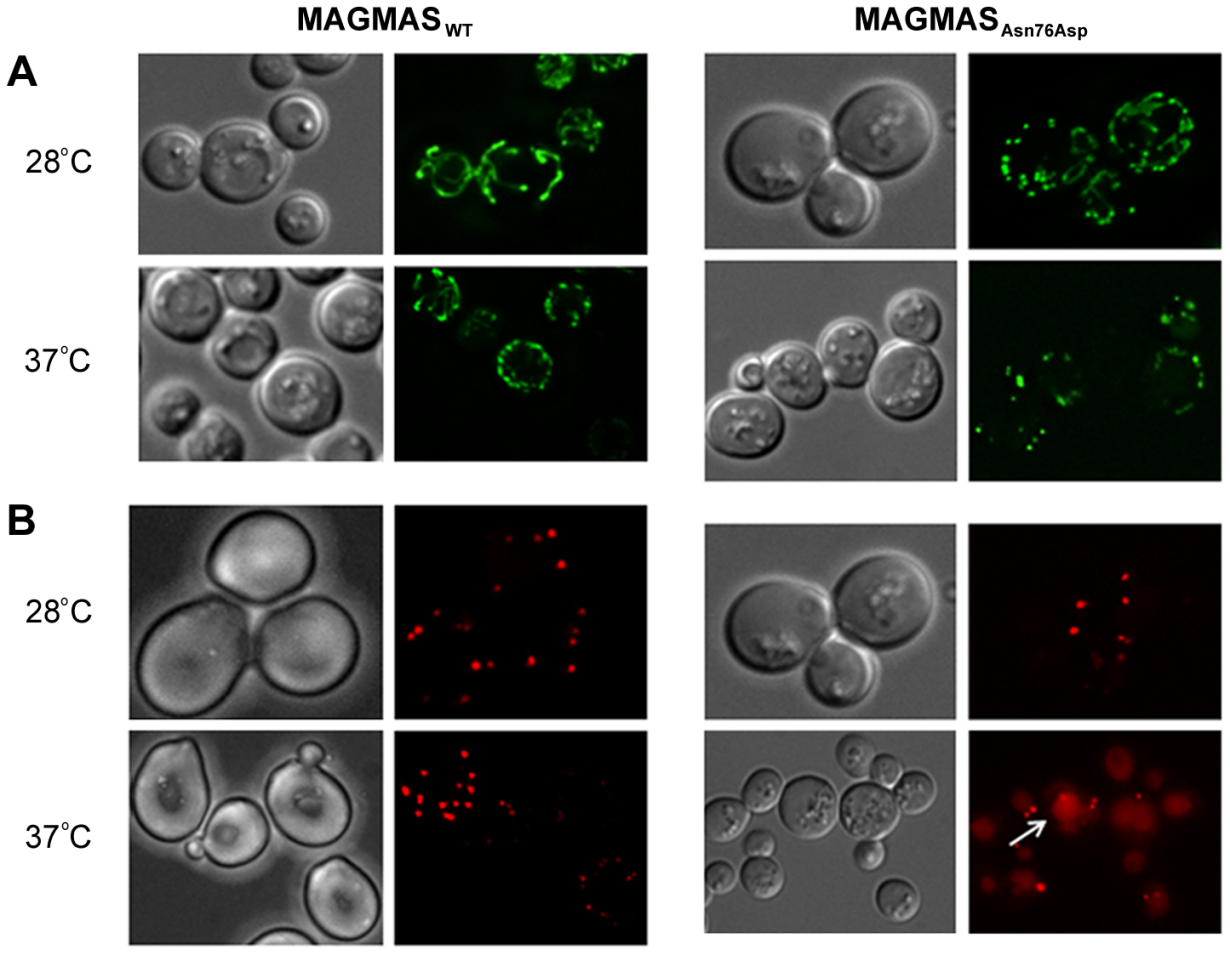
Morphological defects of the yeast *MAGMAS* mutant strain. (**A**) For mitochondria visualization, YPH499-*pam16Δ*-MAG_WT_ and YPH499-*pam16Δ*-MAG_Asn76Asp_ strains were transformed with *pYX232-mtGFP* plasmid; microscopy filters used were 450/490 nm for excitation and 500/550 nm for emission. Unlike the normal reticulated mitochondrial network observed in wild-type *MAGMAS* expressing cells, highly fragmented mitochondria were detected in the mutant strains grown at both permissive and non-permissive temperatures. (**B**) For peroxisomes visualization, BY-*pam16Δ*-MAG_WT_ and BY-*pam16Δ*-MAG***_Asn76Asp_*** were transformed with the pUG34DsRed.SKL reporter plasmid; filters were 542/582 nm for excitation and 604/644 nm for emission. A red halo (white arrow) indicative of pexophagic degradation was observed by mutant cells when grown at the non permissive temperature.

Previous studies in mammalian cells, yeast and drosophila have shown the crucial role of *MAGMAS* and its respective homologs in mitochondria biogenesis [12], 17,20. Altered mitochondria morphology observed in *pam16* yeast mutants and in *Blp*-knowdown drosophila cells led us to study the aspect of these organelles in our mutant strains [20], [21]. Microscopic examination of mitochondria revealed an unhealthy feature in the mutant strain consisting in punctuate and highly fragmented mitochondria that differed from the normal reticulated mitochondrial network observed in WT *MAGMAS* expressing cells. This unusual aspect was more obviously seen in the mutant strains shifted at the non permissive temperature (Figure 8A).

Furthermore, the recent finding of impaired peroxisomes biogenesis in *pam16* yeast mutants [21] prompted us to check the morphology of these organelles in our mutant strains. A remarkable aspect (vacuole labeling), indicative of pexophagic degradation, was only observed in *pam16Δ-*MAG_Asn76Asp_ cells grown at the non permissive temperature compared to *pam16Δ*-MAG_WT_ cells (Figure 8B).

## Discussion

Here, we report the identification of a homozygous missense *MAGMAS* mutation, (p.Asn76Asp), in patients from two unrelated Lebanese families, affected with a rare lethal spondylometaphyseal dysplasia recently described by Mégarbané et al. [6], [7]. By reconstructing haplotypes from exome and STR genotyping data from patients F1-IV.3 and F2-IV.3, we were able to identify a minimal common ancestral homozygous haplotype around *MAGMAS*, spanning 1.9 Mb on chromosome 16p13.3, suggesting the existence of a founder mutation in gene. *MAGMAS*, also referred to as *PAM16*, was first identified in 2001 by Jubinsky et al. [13], as a gene encoding a novel mitochondria-associated protein involved in granulocyte macrophage colony stimulating factor (GM-CSF) signal transduction and expressed by nearly all mammalian cells. The observation of a *MAGMAS* mutation in a severe chondrodysplasia supports a specific role of MAGMAS protein in the endochondral ossification process. Its expression in the notochord and the somites has been reported at day 12.5 embryo and in the cartilage of the developing ribs at day 14.5 embryo [22]. However, its involvement in skeletogenesis has never been described to date. Our findings demonstrate that *MAGMAS* is expressed in trabecular bone and cartilage and more specifically by differentiated chondrocytes localized in the hypertophic zone and by osteoblasts at early developmental stages underlining its key role in skeletogenesis. On the other hand, the implication of the fusion protein CORO7-MAGMAS in the pathogenesis of the skeletal dysplasia has been ruled out, as the related transcript was not well expressed in human fetal chondrocytes and human osteoblasts.

Besides the skeletal features, respiratory insufficiency (F1-IV.1 and F1-IV.3) or heart failure (F2-IV.2 and F2-IV.3) were observed, leading to early deaths of the patients [6], [7]. Of note, an elevated *MAGMAS* level was detected in murine heart and lung in previous studies [22]. These features may also support a key role of MAGMAS in heart and lung.

MAGMAS consists of two main functional domains: a N-terminal hydrophobic region including a membrane association domain (M) and a predicted mitochondria targeting region (T), and a C-terminal J-like domain (J) characterized by three α-helical segments [12], [23]. Importantly, the p.Asn76Asp mutation lies in the helix II of MAGMAS J-like domain. The latter domain forms, with the J-domain of DNAJC19 (ortholog of yeast Pam18 which is another essential component of PAM), a stable heterodimeric subcomplex [19], [24], [25] (Figure S2). Mutations in *DNAJC19* preventing MAGMAS/DNAJC19 dimerization have been shown to lead to dilated cardiomyopathy with ataxia syndrome (DCMA) [26], [27]. Considering the localization of the p.Asn76Asp mutation in the J-like domain of MAGMAS, a similar phenotype was likely to be observed in our reported patients. Cardiomyopathy was observed exclusively in patients of the second family and ataxia was not present but all 4 cases died early before 2 years of age. On the other hand, the absence of DCMA in affected cases may also suggest a redundancy in the function of the subcomplex MAGMAS/DNAJC19 in some tissues.

We further demonstrated that the p.Asn76Asp mutation confers to yeast strains a temperature-sensitive phenotype characterized by a very slow-growth at 34°C (307.15°K) and inviability at 36°C (309.15°K), on both fermentable and non-fermentable growth media. Remarkably, the deleterious effect of the mutation was only observed at a homozygous state which is in correlation with the recessive mode of inheritance of the spondylodysplastic dysplasia reported in this study.

Our findings of impaired import of mitochondrial-matrix proteins in the mutant strains shifted at non-permissive temperature confirmed the deleterious nature of the identified mutation. Likewise, it has been demonstrated that aa alterations in MAGMAS J-like domain resulted in temperature sensitivity and *in vivo* protein import defects in yeast cells [17], [28]. The low expression level of MAGMAS detected in the mutant strain at the non permissive temperature indicates that the p.Asn76Asp mutation confers protein instability. Additionally, we observed unusual punctuated and highly fragmented mitochondria together with enhanced pexophagy in the mutant *MAGMAS* strain at the non permissive temperature, supporting a cell death induction in the yeast mutant strain. These data are consistent with the finding of Roy et al., in *Drosophila* [20] and confirm the crucial role of MAGMAS for cell integrity and viability.

The association between a mitochondrial protein and a skeletal dysplasia is striking as skeletal manifestations are rarely seen in mitochondrial disorders. To date, the only severe skeletal dysplasia identified as having a possible mitochondrial basis is the anauxetic dysplasia [MIM 607095], which is allelic to two milder forms of SD, namely metaphyseal dysplasia Mc Kusick type [MIM 250250] and metaphyseal dysplasia without hypotrichosis [MIM 250460]). The gene responsible for these SD is *RMRP* [MIM 157660], which encodes the RNA component of the mitochondrial RNA processing endoribonuclease (MRP) complex [29]–[31]. Of note, RNase MRP is not exclusively found in the mitochondria but also in the nucleus. Previous studies have shown that mitochondrial and nucleolar RNase MRP have identical RNA components but distinct enzymatic activities and protein components [32]. The recent identification of mutations in *POP1* (Processing of precursor 1) [MIM 602486], which encodes a protein component of the nuclear ribonucleoprotein complex in two siblings with anauxetic dysplasia [33] may support the involvement of the nuclear ribonucleoprotein complex rather than the mitochondrial complex in the etiology of these severe skeletal dysplasias. Similarly, besides its role in preprotein import into mitochondria, Magmas may also be involved in additional important mechanisms as suggested by Short et al. [21]. The detection of different human transcripts of *MAGMAS*, with some lacking the mitochondrial transit peptide (mTP) leader sequence [14] may support a novel extra mitochondrial activity of this protein.

In conclusion, this is the first report linking a mutation in *MAGMAS* with a human developmental disorder and supporting a key role for this mitochondrial protein in the ossification process.

## Materials and Methods

### Patients

Informed consents for participation, sample collection and photographs publication were obtained from both families in compliance with the ethics guidelines of “Conseil de recherche de l'Université Saint-Joseph” (number PTS5). Peripheral blood samples were collected from all available family members.

### DNA extraction and exome sequencing

Genomic DNA was extracted from peripheral blood samples by standard salt-precipitation methods [34]. Exome sequencing was carried-out on 3 individuals from family 2 (F2-III.1, F2-III.2 and F2-IV.3) and the proband from family 1 (individual F1-IV.3) (Figure 2). Targeted exome sequencing, library preparation, capture and sequencing were performed by the French National Genotyping Center (CNG). Exomes were captured and enriched using the in solution Agilent SureSelect Human All Exon kit v3.0 [35] and then sequenced on an Illumina HiSeq2000, using a paired-end 100-bp read sequencing protocol. Image analysis and base calling were performed using the Illumina Data Analysis Pipeline Software 1.5 with default parameters. Raw data were mapped to the current built of the human genome (hg19) by using BWA 0.75 [36]. Variant calling was subsequently performed using GATK 2.5.2 [37] and annotation was done with ANNOVAR [38]. In order to predict the deleterious effect of the identified sequence variations, different bioinformatics tools were applied; such as MutationTaster (http://www.mutationtaster.org/), SIFT (http://sift.bii.a-star.edu.sg/) [9], PROVEAN (http://provean.jcvi.org/index.php/) [10] and PolyPhen-2 (http://genetics.bwh.harvard.edu/pph2/).

### Linkage analysis

Searching for shared homozygous regions using exome data and reconstructing haplotypes were realized using the free software package PLINK (http://pngu.mgh.harvard.edu/purcell/plink/) [11].

### Genotyping of STR markers

The following STR markers flanking the *PAM16* gene and spanning a 8.7 Mb region on chromosome 16p13.3, were amplified using fluorescently labelled primers: D16S521 (AFMA139WG1), D16S3024 (AFMA134XC1), D16S3070 (AFMA33YH1), D16S3027 (AFMA154WC9), D16S758 (CHLC.ATA24C09), D16S3084 (AFMB080YH5), D16S3072 (AFMB015WA9), D16S3134 (AFMA059WF9), D16S510 (AFM312VD5), D16S423 (AFM249YC5), D16S423 (AFM249YC5), AFM339XG1 (RH4035) and D16S3135 (AFMA059XC1). Marker heterozygosity rate ranged from 0.71 (D16S521 and D16S510) to 0.87 (D16S3082 and D16S3027). PCR amplifications were performed in a 30-µl reaction mix, containing 50 ng of genomic DNA, 0.5 units of Taq polymerase (Invitrogen), 1.5 mM MgCl_2_, 5 pmol of each primer, and 0.2 mM of each dNTP. After an initial denaturation step of 5 min at 96°C, 35 cycles of amplification (94°C for 30 sec; 55°C for 30 sec; 72°C for 30 sec) were performed, followed by a final elongation step of 5 min at 72°C. After amplification, PCR products were subjected to denaturing electrophoresis on an ABI PRISM 3100 DNA Sequencer (Applied Biosystems).

### Capillary sequencing

Genomic and cDNA sequences of *MAGMAS* (also known as *PAM16*) were obtained from UCSC Genomic Browser on Human February 2009 (NM_016069). Primers used for PCR amplification were designed using Primer3 software (http://frodo.wi.mit.edu) to amplify the region surrounding the mutation detected by exome sequencing in exon 4. PCR products were purified by exonuclease I/Shrimp Alakaline Phosphatase treatment (ExoSAP-IT; Fisher Scientific SAS, Illkirch, France) according to the manufacturer's instructions and both strands were sequenced using the Big Dye Terminator v1.1 Cycle Sequencing Kit (Applied Biosystems). Sequence reactions were purified on Sephadex G50 (Amersham Pharmacia Biotech, Foster City, CA) and capillary electrophoresis was performed on Genetic Analyser 3100 (Applied Biosystems). Electropherograms were analyzed on the Sequence Analysis Software version 5.2 (Applied Biosystems) and aligned with the WT *MAGMAS* gene sequence using ChromasPro version 1.22 (Technelysium, Queensland, Australia).

### Reverse Transcriptase PCR

Total RNAs were extracted from human primary cultured control cells (fetal chondrocytes, osteoblasts and skin fibroblasts) via the RNeasy Mini Kit (Qiagen). Complementary DNA (cDNA) was synthesized using the SuperScript II Reverse Transcriptase (Invitrogen Life Technologies, Carlsbad, CA, USA) according to the manufacturer's protocol. Sequences of *MAGMAS* (NM_016069), *CORO7* (NM_001201472), *CORO7-MAGMAS* (NM_001201479) and *GAPDH* (NM_002046) were obtained from UCSC Genomic Browser on Human February 2009 and specific primers for cDNA amplification were designed through the Primer3 software as follows:

MAGMAS-Forward: 5′-TACCTGGCCCAGATCATTGT-3′, MAGMAS-Reverse: 5′-GCATCTGCCCTTTTTCTCTG-3′; CORO7-Forward: 5′-GTCCTGGTGTACTGGGCATT-3′, CORO7-Reverse: 5′-AGAGTCAGGGTCCAGCAGAG-3′; CORO7-MAGMAS-Forward: 5′-CTCAGCGCAGTACCTGGAAG-3′, CORO7-MAGMAS-Reverse: 5′-GCCACCCACGGATTTATCAT-3′ and GAPDH-F: 5′-ATGTTCGTCATGGGTGTGAA-3′, GAPDH-R: 5′- TTCAGCTCAGGGATGACCTT-3′.

### Quantitative Real–Time RT-PCR

Reactions were conducted in a 96-well plate with the ABI 7500 Sequence Detection System (Applied Biosystems). Primers against *MAGMAS*, *CORO7* and *CORO7-MAGMAS* were designed using Primer Express software (Applied Biosystems) (Figure S3). PCR was performed in a 20-µl reaction volume containing 10 µl Power SYBR-GreenPCR Master Mix of SYBR Green PCR Master Mix buffer (Applied Biosystems, Foster City, CA, USA), 10 pmol forward and reverse primers and 5 ng of RNAse H-treated (Invitrogen Life Technologies) cDNAs obtained from reverse transcription of DNAse I-treated total RNA extracted from control fibroblasts and chondrocytes cDNA. The reaction cycling conditions were 95°C for 10 min, followed by 40 cycles of 95°C for 15 s and at 60°C for 1 min. Sequence Detection Software was used for exporting the threshold cycle data and further analyzing differences in threshold cycle values (ΔCt) between the test locus and the control locus. The expression values are normalized to that of *GAPDH*. For each gene, the expression ratio for a sample was calculated as the ratio between the average mRNA signal in the sample and the signal from fibroblasts

### Immunohistochemical staining

Femurs isolated from WT mice (at different developmental stages including E 16.5-day, birth and 2 weeks of age) were fixed with 4% paraformaldehyde and embedded in paraffin. Femur sections were stained with Hematoxylin and eosin (H&E) using standard protocol for histological analysis or were subjected to immunohistochemical staining. For immunohistochemistry, sections were stained with Ab specific to MAGMAS (Abcam plc., UK) at 1/200 dilution or with Ab specific to Col X (Quartett) at dilution 1/20 using DAKO EnVision kit. Images were captured with an Olympus PD70- IX2-UCB microscope. All experimental procedures were approved by the French Animal Care and Use Committee.

### Plasmids, yeast strains and growth conditions

Plasmids and yeast strains used in this study are described in table 1. The yeast expression vector *pRS415-WT-MAGMAS*, encoding full-length human WT (WT) *MAGMAS* under the *TEF* promoter was provided by D'Silva *et al.* [17]. The *pRS415-Asn76Asp-MAGMAS* plasmid carrying the point mutation p.Asn76Asp was generated from *pRS415-WT-MAGMAS* through a site directed mutagenesis using the QuickChange protocol (Stratagene) according to the manufacturer's recommendations. Direct sequencing was performed to confirm the successful introduction of the mutation. For construction of yeast strains expressing the human WT/mutated *MAGMAS*, we used *Saccharomyces cerevisiae Δpam16* strains (provided by Delahodde A). For the yeast complementation assay, the diploid strain BY4743 carrying the deleted *pam16* allele (*pam16Δ*::kanMX6) in a heterozygous state was first transformed with *pRS415-WT-MAGMAS* or *pRS415-Asn76Asp-MAGMAS* plasmids, accordingly and then subjected to sporulation and tetrad dissection using standard techniques [39]. Spores from a single ascus were first grown at 28°C (301.15°K) in YPD media then replica-plated to G418 plates for the selection of spores carrying the deleted *pam16* allele and to Leucine drop-out plate for the selection of *pRS415MAGMAS* positive spores. The haploids BY-*pam16Δ*-MAG_WT_ and BY-*pam16Δ*-MAG_Asn76Asp_ were thus generated and spotted onto YPD and YPG to analyze their phenotype. For the construction of YPH499-*pam16Δ*-p*PAM16*-MAG_WT_ and YPH499-*pam16Δ-pPAM16*-MAG_Asn76Asp_, the haploid YPH499*pam16Δ::ADE2* strain containing a plasmid encoding the yeast *PAM16* gene (kindly provided by Guiard B), was transformed either with *pRS415-WT-MAGMAS* or *pRS415-Asn76Asp-MAGMAS* plasmids, respectively then patched onto the selection plates (lacking leucine and uracile). Transformants were subsequently replicated to 5-Fluoroorotic (5-FOA) containing plates, recovered and grown at 28°C (301.15°K), thus generating YPH499-*pam16Δ*-MAG_WT_ and YPH499-*pam16Δ*-MAG_Asn76Asp_ strains that have lost the plasmid containing the yeast *PAM16* gene and the *URA3* marker gene [40]. Ten fold serial dilutions of the different generated strains were subsequently spotted onto YPD plates then incubated at different temperatures for 3 days. Unless otherwise noted, yeast cultures were grown at 28°C (301.15°K). YPD (1% bactopeptone, 1% yeast extract, and 2% glucose) and YPG (1% bactopeptone, 1% yeast extract, and 2% glycerol) were used as rich fermentable and non-fermentable growth media respectively. W0 (0.67% yeast nitrogen base without aa and 2% glucose) supplemented with appropriate nutritional requirements according to the strains was used as a minimal medium. For solid media, 2% bacto agar (Difco) was added.

**Table 1:** Yeast strains and plasmids used in this study.

Yeast strain or Plasmid	Genotype	Source
**BY4743** ***pam16Δ::kanMX6/PAM16***	*leu2Δ/leu2Δ his3Δ/his3Δ ura3Δ/ura3Δ lys2Δ/LYS2 met15Δ/MET15 pam16Δ::kanMX6/PAM16*	Winzeler, E.A. [15]
**YPH499** ***pam16Δ::ADE2-pPAM16***	*leu2Δ ade2Δ ura3Δ his3Δ trp1Δ lys2Δ pam16Δ::ADE2 [pPAM16 URA3]*	Guiard, B. (kind gift)
**BY-** ***pam16Δ*** **-MAG_WT_**	*leu2Δ/leu2Δ his3Δ/his3Δ ura3Δ/ura3Δ lys2Δ/LYS2 met15Δ/MET15 pam16Δ::kanMX6[pRS415-WT-MAGMAS]*	This study
**BY-** ***pam16Δ*** **-MAG_Asn76Asp_**	*leu2Δ/leu2Δ his3Δ/his3Δ ura3Δ/ura3Δ lys2Δ/LYS2 met15Δ/MET15 pam16Δ::kanMX6 [pRS415-Mut-MAGMAS]*	This study
**YPH499-** ***pam16Δ*** **-MAG_WT_**	*leu2Δ ade2Δ ura3Δ his3Δ trp1Δ lys2Δ pam16Δ::ADE2 [pRS415-WT-MAGMAS]*	This study
**YPH499-** ***pam16Δ*** **-MAG_Asn76Asp_**	*leu2Δ ade2Δ ura3Δ his3Δ trp1Δ lys2Δ pam16Δ::ADE2 [pRS415-Mut-MAGMAS]*	This study
**YPH499-** ***pam16Δ*** **-pPAM16-MAG_WT_**	*leu2Δ ade2Δ ura3Δ his3Δ trp1Δ lys2Δ pam16Δ::ADE2 [pPAM16 URA3] [pRS415-WT-MAGMAS]*	This study
**YPH499-** ***pam16Δ*** **-pPAM16-MAG_Asn76Asp_**	*leu2Δ ade2Δ ura3Δ his3Δ trp1Δ lys2Δ pam16Δ::ADE2 [pPAM16 URA3] [pRS415-Mut-MAGMAS]*	This study
***pRS415-WT-MAGMAS***	Amp^R^ *CEN LEU2 TEF MAGMAS_WT_*	D'Silva P. *et al.* [17].
***pRS415-Asn76Asp-MAGMAS***	Amp^R^ *CEN LEU2 TEF MAGMAS_Asn76Asp_*	This study
***pYX232-mtGFP***	*Amp^R^2 micronTP1 preSu9 GFP TRP1*	Westermann B. and Neupert W. [41]
***pUG34 DsRed.SKL***	Amp^R^ *MET25DsRed.SKL HIS3*	Kuravi K.*et al.* [42]

### Protein import and western blotting

For the import activity analysis, YPH499*pam16Δ*-*PAM16*, YPH-*pam16Δ*-MAG_WT_ and YPH-*pam16Δ*-MAG_Asn76Asp_ cells were first grown to OD_600 nm_ of 1.0 at 28°C (301.15°K) in rich medium then shifted to 37°C (310.15°K), for 8 h (28800 s) to induce non-permissive conditions. Cell pellets were suspended in ice-cold TCA buffer (TrisHCl pH 8 20 mM, Ammonium Acetate 50 mM, EDTA 2 mM, protease inhibitor solution, and TCA10%) then lysed by adding glass beads and shaking vigorously for 300 s (5 fold 60 s in ice between). After centrifugation of 600 s (13,000 rpm) at 4°C (277.15°K), the proteins were solubilized in TCA-Laemmli loading buffer (SDS/glycerol stock solution 48%, Tris EDTA 40%, β-mercaptoethanol 0.5%, PMSF 0.2%, protease inhibitor solution 0.2%) and separated by SDS-PAGE. The proteins were subsequently transferred to a nitrocellulose membrane that was first blocked, then incubated 1 h at room temperature with a blocking solution containing the monoclonal HSP60 Ab (provided by Dujardin G.) at 1/15000 dilution. After several washes with TBS1X (Tris Buffered Saline 1X), the membrane was immersed for 1 h at room temperature with HRP – conjugated secondary anti-mouse Ab (at 2/15000 dilution in a blocking solution) then washed again with TBS1X. Secondary antibodies bound to the membrane were detected by chemiluminescence using the Thermo Scientific PierceECL 2 according to the manufacturer's instructions. The same protocol was used for the immunodetection of MAGMAS protein in YPH499, YPH-*pam16Δ*-MAG_WT_ and YPH*-pam16Δ-*MAG_Asn76Asp_strains. For this purpose, we incubated the membrane with a blocking solution containing the polyclonal anti-MAGMAS Ab (Abcam plc., UK) at a 1/1000 final concentration. Therefore, the secondary Ab used in this case was HRP – conjugated secondary anti-rabbit Ab (at 2/15000 dilution in a blocking solution). Phosphoglycerate kinase (PGK) and Porin were detected using anti-PGK (Invitrogen) and anti-Porin (Invitrogen), accordingly.

### Fluorescence microscopy

In order to label mitochondria, YPH-*pam16Δ*-MAG_WT_ and YPH-*pam16Δ*-MAG_Asn76Asp_ strains were transformed with the *pYX232-mtGFP* plasmid that encodes the mitochondrial matrix-targeted GFP [41] and grown for 2 days at 28°C (301.15°K). Cells were secondly incubated overnight at the non-permissive temperature to induce the phenotype. For the visualization of peroxisomes, BY-*pam16Δ-*MAG_WT_ and BY-*pam16Δ-*MAG_Asn76Asp_ were transformed with *pUG34DsRed.SKL* encoding the red fluorescent protein DsRed containing the C-terminal peroxisomal targeting signal type 1 (PTS1, DsRed-SKL). Transformants were grown for 2 days at 28°C (301.15°K) in minimal medium. Harvested cells were washed in Phosphate-buffered saline 1X (PBS1X) then transferred to microscopic slides for imaging. For observations at high temperature, cells grown 2 days at 28°C (301.15°K) in minimal medium, were shifted to 37°C (310.15°K), for 6 hours then harvested, washed and transferred to slides for visualization. The slides were examined with a DMIRE2 microscope (Leica, Deerfield, IL). For GFP fluorescence, filters used were 450/490 nm for excitation and 500/550 nm for emission whereas for Ds.Red fluorescence, filters were 542/582 nm for excitation and 604/644 nm for emission. Images were captured using a CCD camera (Roper Scientific, Tucson, AZ) and Metamorph software (Universal Imaging, WestChester, PA) was used to deconvolute *Z*-series and treat the images.

### Web resources

The URLs for data presented herein are as follows:

Online Mendelian Inheritance in Man (OMIM), http://www.omim.org

Polyphen, http://genetics.bwh.harvard.edu/pph2/

MutationTaster http://www.mutationtaster.org/

SIFT http://sift.bii.a-star.edu.sg/

PROVEAN http://provean.jcvi.org/index.php/

PLINK http://pngu.mgh.harvard.edu/purcell/plink/

Primer3 software http://frodo.wi.mit.edu/

## Supporting Information

Figure S1 Pedigrees and haplotypes of families F1 and F2. Markers are reported from telomere (*top*) to centromere (*bottom*) on chromosome 16p13.3. Blackened symbols represent affected individuals. Disease-bearing chromosome is represented in black. The red box represents the minimal ancestral homozygous haplotype spanning 1.9 Mb between markers D16S758 and D16S243 and shared between F1-IV.3 and F2-IV.3. The normal (A) or mutated (G) allele of *MAGMAS* is indictated within the haplotype.
(TIF)

Figure S2 Protein import pathways into mitochondria. (**A**) Several sophisticated transport machineries mediate recognition, import, sorting and assembly of preproteins into a specific subcompartment of the mitochondria. Preproteins targeted to the matrix cross the outer mitochondrial membrane through the TOM complex then pass through the inner membrane via the TIM23^MOTOR^. Once processed into the matrix, preproteins are folded to their active forms after the cleavage of their presequences by the mitochondrial processing peptidase (MPP). HSP60 assists in the correct folding and assembly of the imported proteins. (**B**) TIM23^MOTOR^: the translocase involved in preproteins import into the mitochondrial matrix. It is composed of TIM23 translocase associated with the PAM complex (In red). The ATP-dependant core of PAM, mtHSP70, drives the translocation and the unfolding of preproteins. The regulation of the position and activity of mtHSP70 is mediated by five other subunits of the PAM complex, consisting of a soluble nucleotide exchange factor MGE1 and four membrane bound co-chaperones TIM44, DNAJC19, PAM16 and PAM17. Via its C-terminal J-like domain, PAM16 (red arrow) interacts with DNAJC19 thus enabling its tethering to the translocon. IM, inner membrane; IMS, intermembrane space; OM: outer membrane.
(TIF)

Figure S3 Primers used for expression analysis by quantitative Real-Time RT-PCR. F: Forward primer. R: Reverse primer. F1, R1 are specific to the *CORO7* transcript; F2, R2 to the *MAGMAS* transcript and F3, R3 to the *CORO7-MAGMAS* transcript.
(TIF)

Table S1 The list of common exonic variations shared at a homozygous state between both probands F1-IV.3 and F2-IV.3.
(DOCX)

## References

[1] Karsenty G, Kronenberg HM, Settembre C (2009) Genetic control of bone formation. Annu Rev Cell Dev Biol 25: 629–648 10.1146/annurev.cellbio.042308.113308 19575648

[2] Baitner AC, Maurer SG, Gruen MB, Di Cesare PE (2000) The genetic basis of the osteochondrodysplasias. J Pediatr Orthop 20: 594–605. 11008738 10.1097/00004694-200009000-00010

[3] Warman ML, Cormier-Daire V, Hall C, Krakow D, Lachman R, et al (2011) Nosology and classification of genetic skeletal disorders: 2010 revision. Am J Med Genet A 155A: 943–968 10.1002/ajmg.a.33909 21438135 PMC3166781

[4] Ikegawa S (2006) Genetic analysis of skeletal dysplasia: recent advances and perspectives in the post-genome-sequence era. J Hum Genet 51: 581–586 10.1007/s10038-006-0401-x 16670815

[5] Huber C, Faqeih EA, Bartholdi D, Bole-Feysot C, Borochowitz Z, et al (2013) Exome sequencing identifies INPPL1 mutations as a cause of opsismodysplasia. Am J Hum Genet 92: 144–149 10.1016/j.ajhg.2012.11.015 23273569 PMC3542463

[6] Mégarbané A, Dagher R, Melki I (2008) Sib pair with previously unreported skeletal dysplasia. Am J Med Genet A 146A: 2916–2919 10.1002/ajmg.a.32540 18925669

[7] Mégarbané A, Mehawej C, El Zahr A, Haddad S, Cormier-Daire V (2013) A Second Family with Autosomal Recessive Spondylometaphyseal Dysplasia and Early Death. Am J Med Genet A 164: 1010–4 10.1002/ajmg.a.36372 24458487

[8] Lander ES, Botstein D (1987) Homozygosity mapping: a way to map human recessive traits with the DNA of inbred children. Science 236: 1567–1570. 2884728 10.1126/science.2884728

[9] Kumar P, Henikoff S, Ng PC (2009) Predicting the effects of coding non-synonymous variants on protein function using the SIFT algorithm. Nat Protoc 4: 1073–1081 10.1038/nprot.2009.86 19561590

[10] Choi Y, Sims GE, Murphy S, Miller JR, Chan AP (2012) Predicting the functional effect of amino acid substitutions and indels. PloS One 7: e46688 10.1371/journal.pone.0046688 23056405 PMC3466303

[11] Purcell S, Neale B, Todd-Brown K, Thomas L, Ferreira MAR, et al (2007) PLINK: a tool set for whole-genome association and population-based linkage analyses. Am J Hum Genet 81: 559–575 10.1086/519795 17701901 PMC1950838

[12] Frazier AE, Dudek J, Guiard B, Voos W, Li Y, et al (2004) Pam16 has an essential role in the mitochondrial protein import motor. Nat Struct Mol Biol 11: 226–233 10.1038/nsmb735 14981507

[13] Jubinsky PT, Messer A, Bender J, Morris RE, Ciraolo GM, et al (2001) Identification and characterization of Magmas, a novel mitochondria-associated protein involved in granulocyte-macrophage colony-stimulating factor signal transduction. Exp Hematol 29: 1392–1402. 11750097 10.1016/s0301-472x(01)00749-4

[14] Peng J, Huang C-H, Short MK, Jubinsky PT (2005) Magmas gene structure and evolution. In Silico Biol 5: 251–263. 15984936

[15] Winzeler EA, Shoemaker DD, Astromoff A, Liang H, Anderson K, et al (1999) Functional characterization of the S. cerevisiae genome by gene deletion and parallel analysis. Science 285: 901–906. 10436161 10.1126/science.285.5429.901

[16] Becker S, Gehrsitz A, Bork P, Buchner S, Buchner E (2001) The black-pearl gene of Drosophila defines a novel conserved protein family and is required for larval growth and survival. Gene 262: 15–22 10.1016/S0378-1119(00)00548-5 11179663

[17] Sinha D, Joshi N, Chittoor B, Samji P, D'Silva P (2010) Role of Magmas in protein transport and human mitochondria biogenesis. Hum Mol Genet 19: 1248–1262 10.1093/hmg/ddq002 20053669 PMC2838536

[18] Schatz G, Dobberstein B (1996) Common principles of protein translocation across membranes. Science 271: 1519–1526. 8599107 10.1126/science.271.5255.1519

[19] Wagner K, Mick DU, Rehling P (2009) Protein transport machineries for precursor translocation across the inner mitochondrial membrane. Biochim Biophys Acta 1793: 52–59 10.1016/j.bbamcr.2008.05.026 18590776

[20] Roy S, Short MK, Stanley ER, Jubinsky PT (2012) Essential role of Drosophila black-pearl is mediated by its effects on mitochondrial respiration. FASEB J Off Publ Fed Am Soc Exp Biol 26: 3822–3833 10.1096/fj.11-193540 PMC3425817 22700875

[21] Short MK, Hallett JP, Tar K, Dange T, Schmidt M, et al (2012) The yeast magmas ortholog pam16 has an essential function in fermentative growth that involves sphingolipid metabolism. PloS One 7: e39428 10.1371/journal.pone.0039428 22808036 PMC3393719

[22] Jubinsky PT, Short MK, Mutema G, Witte DP (2003) Developmental expression of Magmas in murine tissues and its co-expression with the GM-CSF receptor. J Histochem Cytochem Off J Histochem Soc 51: 585–596. 10.1177/002215540305100504 12704206

[23] D'Silva PR, Schilke B, Walter W, Craig EA (2005) Role of Pam16's degenerate J domain in protein import across the mitochondrial inner membrane. Proc Natl Acad Sci U S A 102: 12419–12424 10.1073/pnas.0505969102 16105940 PMC1194952

[24] Elsner S, Simian D, Iosefson O, Marom M, Azem A (2009) The mitochondrial protein translocation motor: Structural conservation between the human and yeast Tim14/Pam18-Tim16/Pam16 co-chaperones. Int J Mol Sci 10: 2041–2053 10.3390/ijms10052041 19564938 PMC2695266

[25] Chacinska A, Koehler CM, Milenkovic D, Lithgow T, Pfanner N (2009) Importing mitochondrial proteins: machineries and mechanisms. Cell 138: 628–644 10.1016/j.cell.2009.08.005 19703392 PMC4099469

[26] Sparkes R, Patton D, Bernier F (2007) Cardiac features of a novel autosomal recessive dilated cardiomyopathic syndrome due to defective importation of mitochondrial protein. Cardiol Young 17: 215–217 10.1017/S1047951107000042 17244376

[27] Davey KM, Parboosingh JS, McLeod DR, Chan A, Casey R, et al (2006) Mutation of DNAJC19, a human homologue of yeast inner mitochondrial membrane co-chaperones, causes DCMA syndrome, a novel autosomal recessive Barth syndrome-like condition. J Med Genet 43: 385–393 10.1136/jmg.2005.036657 16055927 PMC2564511

[28] D'Silva PR, Schilke B, Hayashi M, Craig EA (2007) Interaction of the J-Protein Heterodimer Pam18/Pam16 of the Mitochondrial Import Motor with the Translocon of the Inner Membrane. Mol Biol Cell 19: 424–432 10.1091/mbc.E07-08-0748 18003975 PMC2174176

[29] Thiel CT, Horn D, Zabel B, Ekici AB, Salinas K, et al (2005) Severely incapacitating mutations in patients with extreme short stature identify RNA-processing endoribonuclease RMRP as an essential cell growth regulator. Am J Hum Genet 77: 795–806 10.1086/497708 16252239 PMC1271388

[30] Ridanpää M, van Eenennaam H, Pelin K, Chadwick R, Johnson C, et al (2001) Mutations in the RNA component of RNase MRP cause a pleiotropic human disease, cartilage-hair hypoplasia. Cell 104: 195–203. 11207361 10.1016/s0092-8674(01)00205-7

[31] Bonafé L, Schmitt K, Eich G, Giedion A, Superti-Furga A (2002) RMRP gene sequence analysis confirms a cartilage-hair hypoplasia variant with only skeletal manifestations and reveals a high density of single-nucleotide polymorphisms. Clin Genet 61: 146–151. 11940090 10.1034/j.1399-0004.2002.610210.x

[32] Lu Q, Wierzbicki S, Krasilnikov AS, Schmitt ME (2010) Comparison of mitochondrial and nucleolar RNase MRP reveals identical RNA components with distinct enzymatic activities and protein components. RNA N Y N 16: 529–537 10.1261/rna.1893710 PMC2822918 20086051

[33] Glazov EA, Zankl A, Donskoi M, Kenna TJ, Thomas GP, et al (2011) Whole-exome re-sequencing in a family quartet identifies POP1 mutations as the cause of a novel skeletal dysplasia. PLoS Genet 7: e1002027 10.1371/journal.pgen.1002027 21455487 PMC3063761

[34] Miller SA, Dykes DD, Polesky HF (1988) A simple salting out procedure for extracting DNA from human nucleated cells. Nucleic Acids Res 16: 1215. 3344216 10.1093/nar/16.3.1215 PMC334765

[35] Sulonen A-M, Ellonen P, Almusa H, Lepistö M, Eldfors S, et al (2011) Comparison of solution-based exome capture methods for next generation sequencing. Genome Biol 12: R94 10.1186/gb-2011-12-9-r94 21955854 PMC3308057

[36] Li H, Durbin R (2009) Fast and accurate short read alignment with Burrows-Wheeler transform. Bioinforma Oxf Engl 25: 1754–1760 10.1093/bioinformatics/btp324 PMC2705234 19451168

[37] McKenna A, Hanna M, Banks E, Sivachenko A, Cibulskis K, et al (2010) The Genome Analysis Toolkit: a MapReduce framework for analyzing next-generation DNA sequencing data. Genome Res 20: 1297–1303 10.1101/gr.107524.110 20644199 PMC2928508

[38] Wang K, Li M, Hakonarson H (2010) ANNOVAR: functional annotation of genetic variants from high-throughput sequencing data. Nucleic Acids Res 38: e164 10.1093/nar/gkq603 20601685 PMC2938201

[39] Schiestl RH, Gietz RD (1989) High efficiency transformation of intact yeast cells using single stranded nucleic acids as a carrier. Curr Genet 16: 339–346. 2692852 10.1007/BF00340712

[40] Sikorski RS, Boeke JD (1991) In vitro mutagenesis and plasmid shuffling: from cloned gene to mutant yeast. Methods Enzymol 194: 302–318. 2005795 10.1016/0076-6879(91)94023-6

[41] Westermann B, Neupert W (2000) Mitochondria-targeted green fluorescent proteins: convenient tools for the study of organelle biogenesis in Saccharomyces cerevisiae. Yeast 16: 1421–1427 10.1002/1097-0061(200011)16:15%3C1421::AID-YEA624%3E3.0.CO;2-U 11054823

[42] Kuravi K, Nagotu S, Krikken AM, Sjollema K, Deckers M, et al (2006) Dynamin-related proteins Vps1p and Dnm1p control peroxisome abundance in Saccharomyces cerevisiae. J Cell Sci 119: 3994–4001 10.1242/jcs.03166 16968746

