# Supplementary figures and images for: The Impairment of MAGMAS Function in Human Is Responsible for a Severe Skeletal Dysplasia
Source: PLoS Genet. 2014 May 1;10(5):e1004311. doi: 10.1371/journal.pgen.1004311 (PMC4006740; doi:10.1371/journal.pgen.1004311)

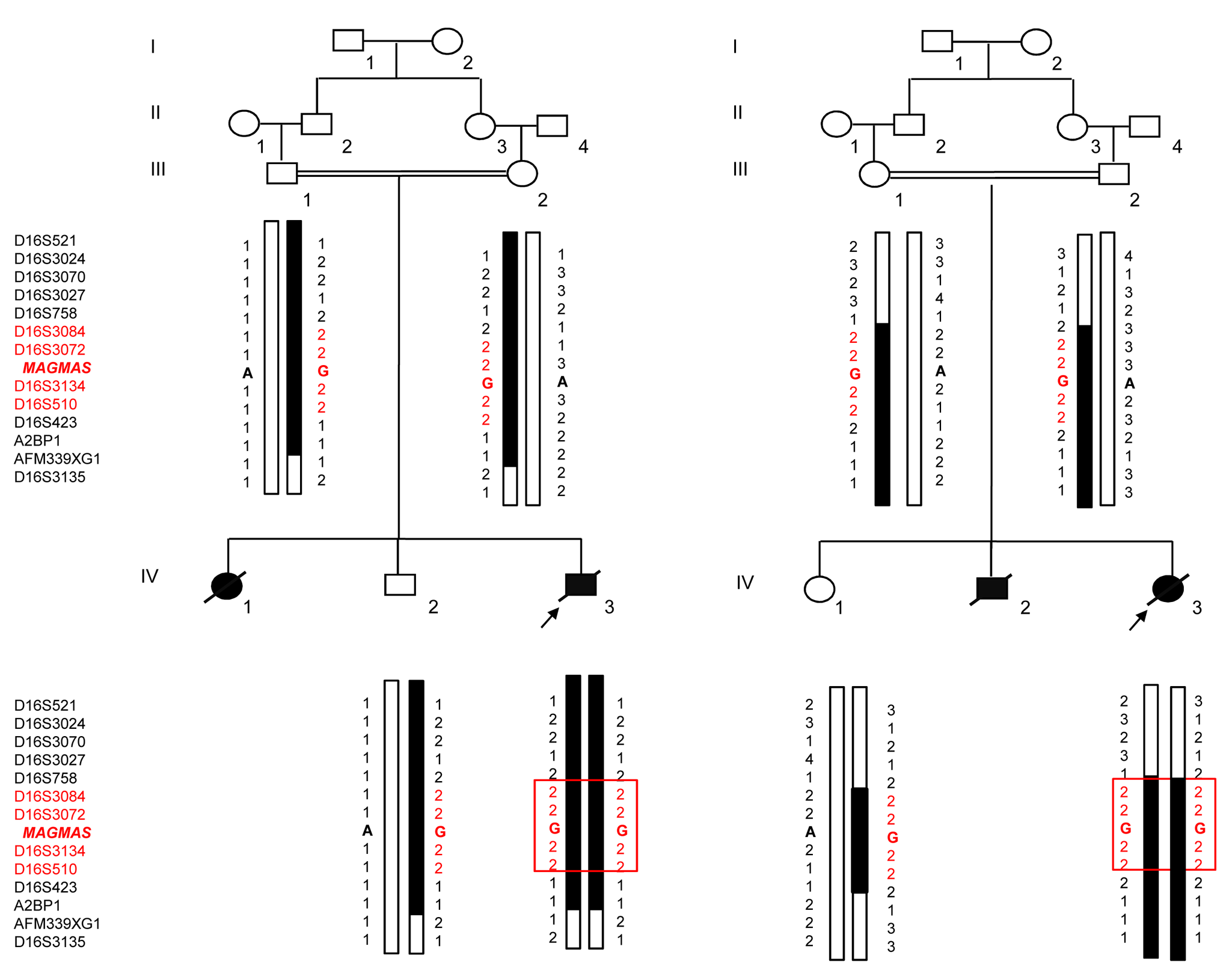

Supplement: Figure S1 — Pedigrees and haplotypes of families F1 and F2. Markers are reported from telomere (top) to centromere (bottom) on chromosome 16p13.3. Blackened symbols represent affected individuals. Disease-bearing chromosome is represented in black. The red box represents the minimal ancestral homozygous haplotype spanning 1.9 Mb between markers D16S758 and D16S243 and shared between F1-IV.3 and F2-IV.3. The normal (A) or mutated (G) allele of MAGMAS is indictated within the haplotype. (TIF) [file pgen.1004311.s001.tif]

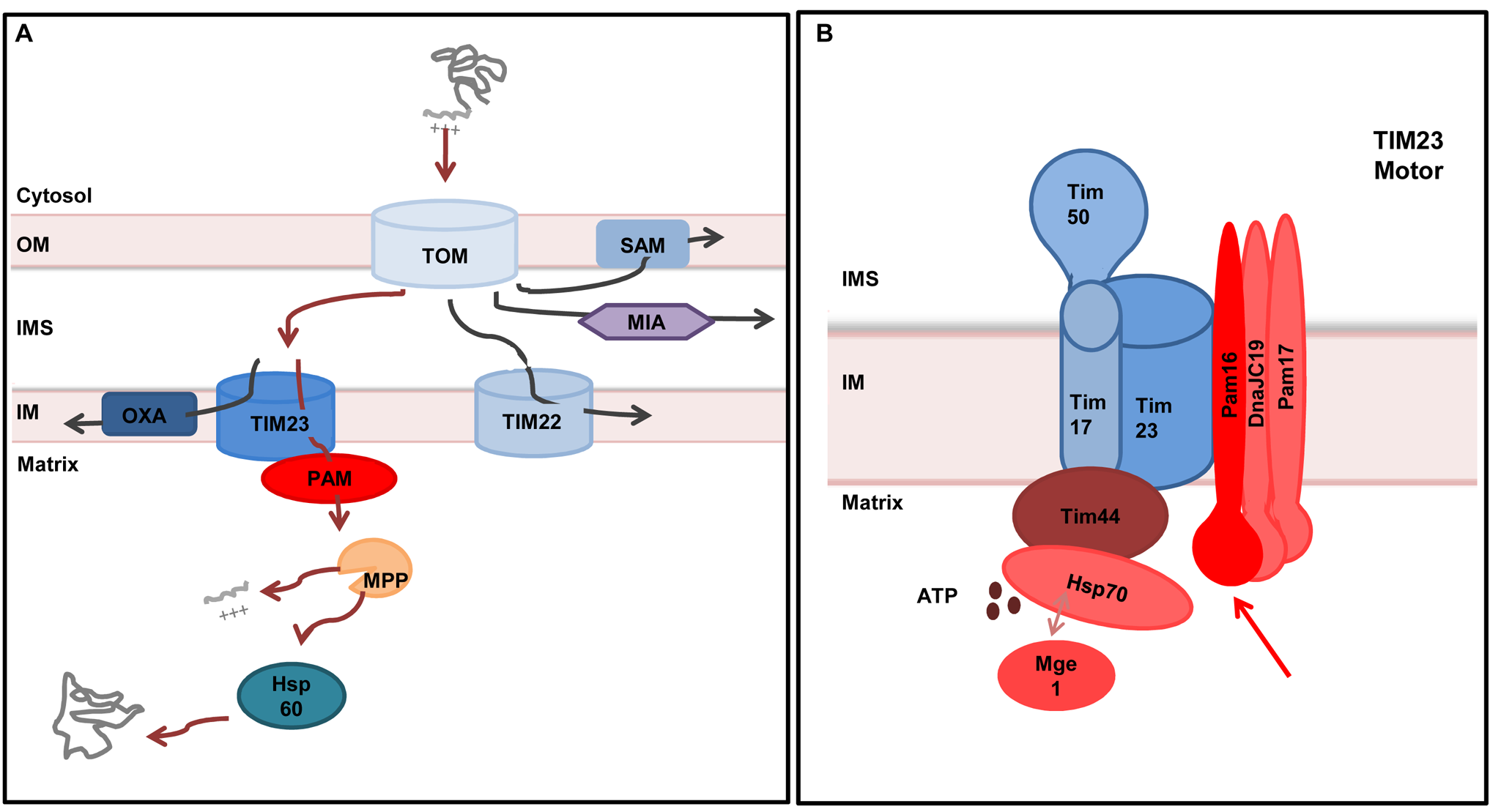

Supplement: Figure S2 — Protein import pathways into mitochondria. (A) Several sophisticated transport machineries mediate recognition, import, sorting and assembly of preproteins into a specific subcompartment of the mitochondria. Preproteins targeted to the matrix cross the outer mitochondrial membrane through the TOM complex then pass through the inner membrane via the TIM23MOTOR. Once processed into the matrix, preproteins are folded to their active forms after the cleavage of their presequences by the mitochondrial processing peptidase (MPP). HSP60 assists in the correct folding and assembly of the imported proteins. (B) TIM23MOTOR: the translocase involved in preproteins import into the mitochondrial matrix. It is composed of TIM23 translocase associated with the PAM complex (In red). The ATP-dependant core of PAM, mtHSP70, drives the translocation and the unfolding of preproteins. The regulation of the position and activity of mtHSP70 is mediated by five other subunits of the PAM complex, consisting of a soluble nucleotide exchange factor MGE1 and four membrane bound co-chaperones TIM44, DNAJC19, PAM16 and PAM17. Via its C-terminal J-like domain, PAM16 (red arrow) interacts with DNAJC19 thus enabling its tethering to the translocon. IM, inner membrane; IMS, intermembrane space; OM: outer membrane. (TIF) [file pgen.1004311.s002.tif]

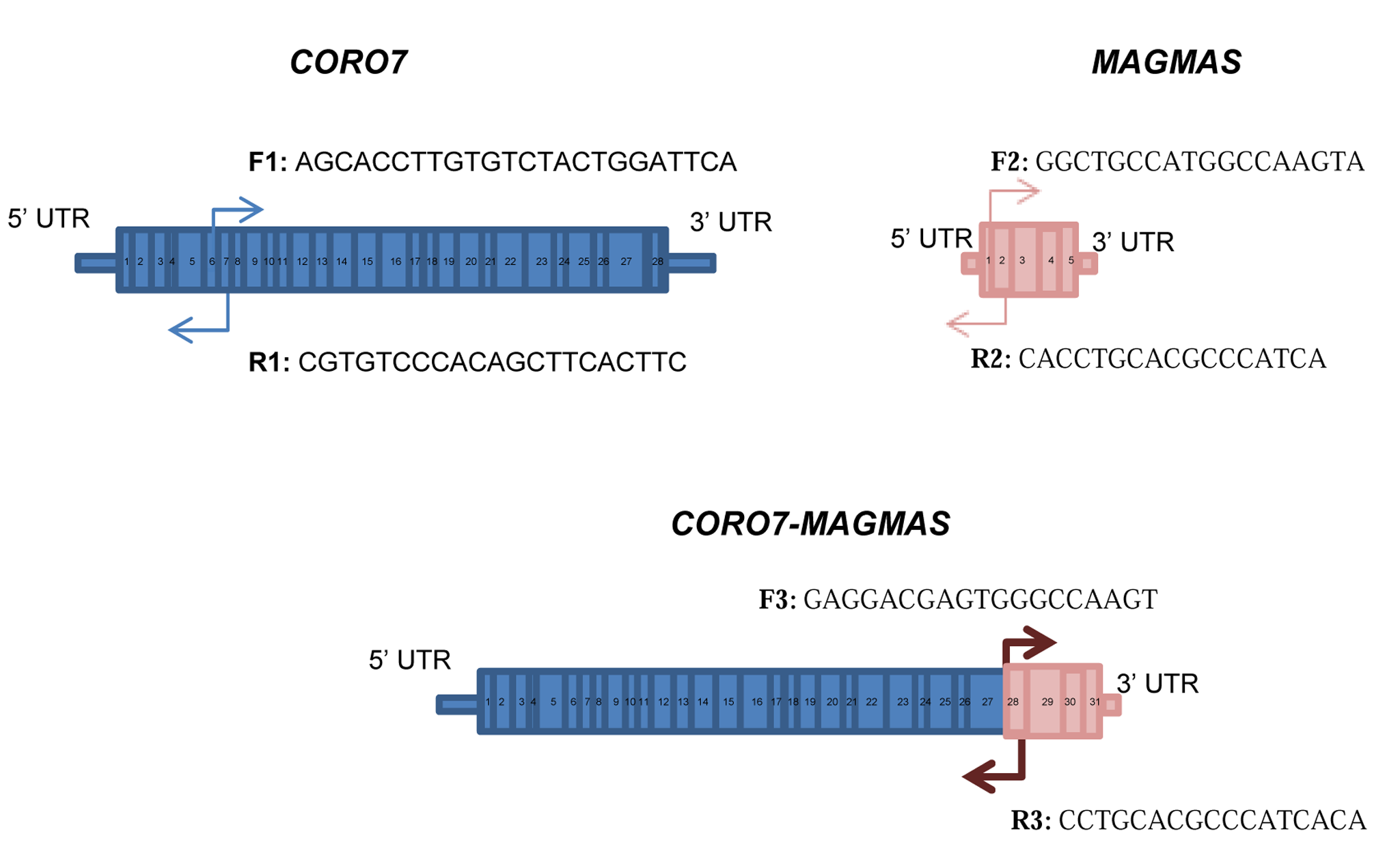

Supplement: Figure S3 — Primers used for expression analysis by quantitative Real-Time RT-PCR. F: Forward primer. R: Reverse primer. F1, R1 are specific to the CORO7 transcript; F2, R2 to the MAGMAS transcript and F3, R3 to the CORO7-MAGMAS transcript. (TIF) [file pgen.1004311.s003.tif]
