# Supplementary material for: The Impairment of MAGMAS Function in Human Is Responsible for a Severe Skeletal Dysplasia
Source: PLoS Genet. 2014 May 1;10(5):e1004311. doi: 10.1371/journal.pgen.1004311 (PMC4006740; doi:10.1371/journal.pgen.1004311)
Supplement: Table S1 — The list of common exonic variations shared at a homozygous state between both probands F1-IV.3 and F2-IV.3. (DOCX) [file pgen.1004311.s004.docx]

**Supplementary Table S1:** The list of common exonic variations shared at a homozygous state between both probands F1-IV.3 and F2-IV.3. SNV: Single Nucleotide Variation.

| Chr | Start | End | Gene | Function | Exonic Function | Amino Acid change |
| --- | --- | --- | --- | --- | --- | --- |
| Chr9 | 88937852 | 88937854 | ZCCHC6 | Exonic | nonframeshift deletion | ZCCHC6:NM_001185074:exon9: c.2442_2444del:p.814_815del, ZCCHC6:NM_001185059:exon13 c.2811_2813del:p.937_938del,ZCCHC6:NM_024617:exon13:c.2811_2813del:p.937_938del |
| Chr16 | 4390986 | 4390986 | PAM16, CORO7-PAM16 | Exonic | nonsynonymous SNV | PAM16:NM_016069:exon4:c.A226G:p.N76D, CORO7-PAM16:NM_001201479: exon30 :c.A2995G:p.N999D |
| Chr21 | 31913982 | 31913982 | KRTAP19-6 | Exonic | frameshift deletion | KRTAP19-6: NM_181612:exon1:c.171delC:p.F57fs |
